# State Laws Regarding Indoor Public Use, Retail Sales, and Prices of Electronic Cigarettes — U.S. States, Guam, Puerto Rico, and U.S. Virgin Islands, September 30, 2017

**DOI:** 10.15585/mmwr.mm6649a1

**Published:** 2017-12-15

**Authors:** Kristy Marynak, Brandon Kenemer, Brian A. King, Michael A. Tynan, Allison MacNeil, Elizabeth Reimels

**Affiliations:** 1 Office on Smoking and Health, National Center for Chronic Disease Prevention and Health Promotion, CDC.

Electronic cigarettes (e-cigarettes) are the most frequently used tobacco product among U.S. youths, and past 30-day e-cigarette use is more prevalent among high school students than among adults (*1*,*2*). E-cigarettes typically deliver nicotine, and the U.S. Surgeon General has concluded that nicotine exposure during adolescence can cause addiction and can harm the developing adolescent brain (*2*). Through authority granted by the Family Smoking Prevention and Tobacco Control Act, the Food and Drug Administration (FDA) prohibits e-cigarette sales to minors, free samples, and vending machine sales, except in adult-only facilities (*3*). States, localities, territories, and tribes maintain broad authority to adopt additional or more stringent requirements regarding tobacco product use, sales, marketing, and other topics (*2*,*4*). To understand the current e-cigarette policy landscape in the United States, CDC assessed state and territorial laws that 1) prohibit e-cigarette use and conventional tobacco smoking indoors in restaurants, bars, and worksites; 2) require a retail license to sell e-cigarettes; 3) prohibit e-cigarette self-service displays (e.g., requirement that products be kept behind the counter or in a locked box); 4) establish 21 years as the minimum age of purchase for all tobacco products, including e-cigarettes (tobacco-21); and 5) apply an excise tax to e-cigarettes. As of September 30, 2017, eight states, the District of Columbia (DC), and Puerto Rico prohibited indoor e-cigarette use and smoking in indoor areas of restaurants, bars, and worksites; 16 states, DC, and the U.S. Virgin Islands required a retail license to sell e-cigarettes; 26 states prohibited e-cigarette self-service displays; five states, DC, and Guam had tobacco-21 laws; and eight states, DC, Puerto Rico, and the U.S. Virgin Islands taxed e-cigarettes. Sixteen states had none of the assessed laws. A comprehensive approach that combines state-level strategies to reduce youths’ initiation of e-cigarettes and population exposure to e-cigarette aerosol, coupled with federal regulation, could help reduce health risks posed by e-cigarettes among youths (*2*,*5*).

Effective and enacted dates for laws enacted as of September 30, 2017, were obtained from the CDC State Tobacco Activities Tracking and Evaluation (STATE) System for the 50 states, DC, Puerto Rico, the U.S. Virgin Islands, and Guam.* Legislation information is collected quarterly from the Westlaw online legal research database and is analyzed, coded, and entered into STATE by CDC.^†^ State laws and regulations prohibiting self-service displays of e-cigarettes were obtained from the Tobacco Control Legal Consortium (*6*); effective and enacted dates and territory laws were reviewed in the Westlaw database and on territory websites.

As of September 30, 2017, eight states, DC, and Puerto Rico prohibited indoor e-cigarette use and conventional tobacco smoking in worksites, restaurants, and bars (Figure 1). E-cigarette self-service display restrictions were the most commonly enacted of the five types of laws (26 states), followed by retail license requirements (16 states, DC, and the U.S. Virgin Islands) (Table). Tobacco-21 was the least common law, taking effect in California, Hawaii, and DC in 2016; in Maine, New Jersey, and Oregon in 2017; and in Guam in 2018. Eight states, DC, Puerto Rico, and the U.S. Virgin Islands taxed e-cigarettes, with approaches varying by state. Five of these tax laws have been adjusted since enactment: California, Minnesota, and the U.S. Virgin Islands increased the tax rate, and Kansas and DC decreased the tax rate.

**FIGURE 1:**
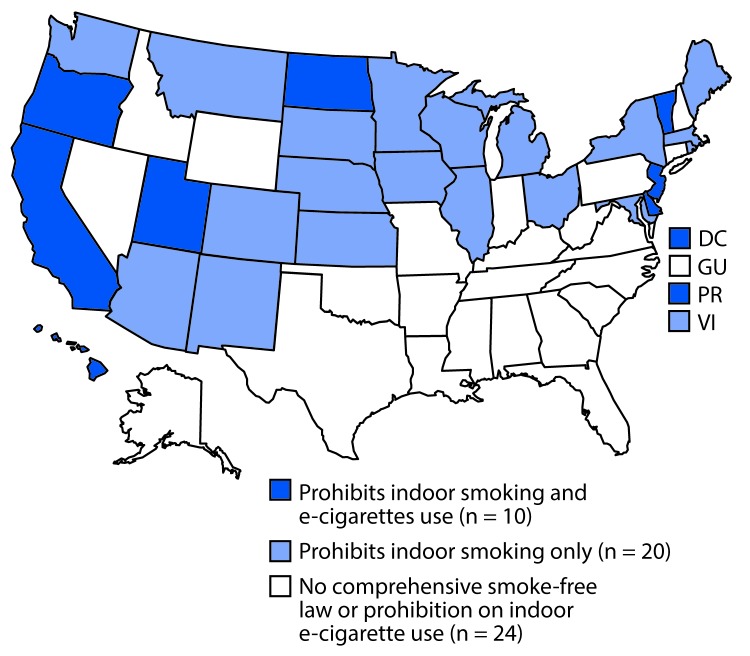
States and territories with and without laws* prohibiting smoking and use of e-cigarettes in indoor areas of private worksites, restaurants, and bars — United States, September 30, 2017 **Abbreviations:** DC = District of Columbia; GU = Guam; PR = Puerto Rico; VI = U.S. Virgin Islands. *A comprehensive state smoke-free law is defined as one that prohibits smoking in indoor areas of private worksites, restaurants, and bars.

**TABLE:** State laws regarding indoor public use, retail sales, and prices of electronic cigarettes — U.S. states and Guam, Puerto Rico, and U.S. Virgin Islands, enacted as of September 30, 2017

State/Territory	Effective date					Summary of laws^†^ enacted as of September 30, 2017
	Prohibits e-cigarette use in worksites, restaurants, and bars	Retail license required to sell e-cigarettes over the counter	Self-service displays of e-cigarettes prohibited*	Sales of tobacco products including e-cigarettes to persons aged <21 yrs prohibited	E-cigarette tax (tax rate)	
Alabama	—^§^	—	—	—	—	—
Alaska	—	—	—	—	—	—
Arizona	—	—	—	—	—	—
Arkansas	—	May 1, 2015	Jul 22, 2015	—	—	RL, SS
California	Jun 9, 2016	Jan 1, 2017	Jun 9, 2016	Jun 9, 2016^¶^	4/1/2017; (27.3% wholesale cost) 7/1/2017; (65.08% wholesale cost)**	EF, RL, SS, T-21, T
Colorado	—	—	—	—	—	—
Connecticut	—	Mar 1, 2016	—	—	—	RL
Delaware	Oct 5, 2015	—	Jun 12, 2014	—	1/1/2018; $0.05 per fluid mL	EF, SS, T
District of Columbia	Nov 18,2016	Oct 22, 2015	—	Nov 29, 2016	10/1/2015 (67% wholesale sales price) 10/1/2016 (65% wholesale sales price)**	EF, RL, T-21, T
Florida	—	—	Jul 1, 2014	—	—	SS
Georgia	—	—	—	—	—	—
Guam	—	—	—	Jan 1, 2018^††^	—	T-21
Hawaii	Jan 1, 2016	—	Jul 1, 2014	Jan 1, 2016	—	EF, SS, T-21
Idaho	—	—	Jul 1, 2012	—	—	SS
Illinois	—	—	Jan 1, 2015	—	—	SS
Indiana	—	Jul 1, 2015	Jul 1, 2013	—	—	RL, SS
Iowa	—	Jul 1, 2014	Jul 1, 2014	—	—	RL, SS
Kansas	—	Jul 1, 2012	Jul 1, 2012	—	Jan 1, 2017 ($0.20 per mL of consumable material) Jul 1, 2017 ($0.05 per mL of consumable material)**	RL, SS, T
Kentucky	—	—	—	—	—	—
Louisiana	—	May 28, 2014	May 5, 2014	—	Jul 1, 2015 ($0.05 per liquid mL of nicotine)	RL, SS, T
Maine	—	Nov 1, 2017	Mar 3, 2016*	Nov 1, 2017^§§^	—	RL, SS, T-21
Maryland	—	Oct 1, 2017	—	—	—	RL
Massachusetts	—	—	Sep 25, 2015*	—	—	SS
Michigan	—	—	—	—	—	—
Minnesota	—	Aug 1, 2014	Jul 1, 2014	—	Aug 1, 2010 (35% wholesale sales price) Jul 1, 2013 (95% wholesale sales price)**	RL, SS, T
Mississippi	—	—	—	—	—	—
Missouri	—	—	—	—	—	—
Montana	—	Jan 1, 2016	—	—	—	RL
Nebraska	—	—	Feb 27, 2015	—	—	SS
Nevada	—	—	—	—	—	—
New Hampshire	—	—	—	—	—	—
New Jersey	Jul 11, 2010	—	—	Nov 1, 2017	—	EF, T-21
New Mexico	—	—	Jun 19, 2015	—	—	SS
New York	—	—	Dec 29, 2014	—	—	SS
North Carolina	—	—	—	—	Jun 1, 2015 ($0.05 per fluid mL)	T
North Dakota	Dec 6, 2012	—	Aug 1, 2015	—	—	EF, SS
Ohio	—	—	—	—	—	—
Oklahoma	—	—	Nov 1, 2014	—	—	SS
Oregon	Jan 1, 2016	—	May 26, 2015	Aug 9, 2017	—	EF, SS, T-21
Pennsylvania	—	Jul 13, 2016	—	—	Jul 13, 2016 (40% purchase price)	RL, T
Puerto Rico	Apr 11, 2011	—	—	—	May 29, 2017 ($3.00 per e-cigarette)	EF, T
Rhode Island	—	Jan 1, 2015	—	—	—	RL
South Carolina	—	—	—	—	—	—
South Dakota	—	—	Jul 1, 2014	—	—	SS
Tennessee	—	—	—	—	—	—
Texas	—	—	Oct 1, 2015	—	—	SS
U.S. Virgin Islands	—	May 16, 2014	—	—	Oct 15, 2014 (20% cost price) Mar 23, 2016 (45% cost price)**	RL, T
Utah	May 8, 2012	Jul 1, 2015	Jul 1, 2015	—	—	EF, RL, SS
Vermont	Jul 1, 2016	Jul 1, 2013	Jan 1, 2017	—	—	EF, RL, SS
Virginia	—	—	—	—	—	—
Washington	—	Jun 28, 2016	Jun 28, 2016	—	—	RL, SS
West Virginia	—	—	—	—	Jul 1, 2016 ($0.075 per fluid mL)	T
Wisconsin	—	—	—	—	—	—
Wyoming	—	—	Mar 13, 2013	—	—	SS
**Total**	**8 states, DC, and Puerto Rico**	**16 states, DC, and U.S Virgin Islands**	**26 states**	**5 states, DC, and Guam**	**8 states, DC, Puerto Rico and U.S. Virgin Islands**	**—**

**Abbreviations:** EF = E-cigarette free indoor air law; RL = retail license; SS = self-service; T = excise tax; T-21 = sales to persons aged <21 years prohibited.

* Self-service display laws include regulations for Maine and Massachusetts, as reviewed by the Tobacco Control Legal Consortium.

^†^ EF: state law prohibits e-cigarette use in indoor areas of private worksites, restaurants, and bars; RL: state law requires retailer to purchase a license to sell e-cigarettes; SS: state law prohibits self-service displays of e-cigarettes; T: state law applies tax to e-cigarettes; T-21: state law prohibits sales of tobacco products, including e-cigarettes, to persons aged <21 years.

^§^ Dashes indicate that laws related to these topics were not accessed for this state.

^¶^ In California, the law prohibiting sales to persons <21 years of age does not apply to the sale, giving, or furnishing of tobacco products to active duty military personnel who are aged ≥18 years.

** In California, District of Columbia, Kansas, Minnesota, and U.S. Virgin Islands, legislation was updated to reflect changes in the excise tax rates. The effective dates presented represent the original and updated laws.

^††^ Guam’s T-21 law has been enacted but will not take effect until 2018.

^§§ ^ Maine’s provisions for raising the minimum age of sale of tobacco to 21 years will not begin to be enforced until July 2018. In addition, persons who had attained 18 years of age as of July 1, 2018, will continue to be allowed to buy tobacco products.

The number of newly enacted laws increased from four to 16 during 2013–2014 and from 16 to 21 during 2014–2015, but decreased from 21 to 15 during 2015–2016. Eight laws were enacted during January–September 2017 (Figure 2). A total of 72 laws were enacted in 34 states, DC, and three territories during January 2010–September 2017. Sixteen states did not have any of the five assessed laws, and California was the only state with all five of the assessed laws.

**FIGURE 2:**
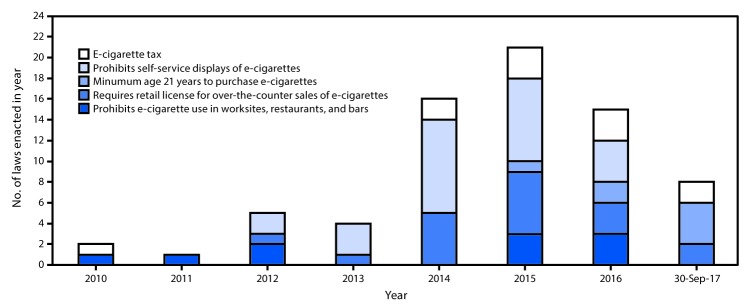
Number of state and territorial* laws that address indoor use, retail sales, and prices of e-cigarettes, enacted as of September 30, 2017^†^ — United States, 2010–2017 * Guam, Puerto Rico, and U.S. Virgin Islands. ^†^ In California, District of Columbia, Kansas, Minnesota, and U.S. Virgin Islands, legislation was updated in later years to reflect changes in tax rates. To avoid duplication, this figure presents the enacted dates only of the original law.

## Discussion

Several states have enacted laws related to e-cigarettes in recent years, ranging from tobacco-21 laws in five states, DC, and Guam, to self-service display restrictions in approximately half of the states. Legislative activity increased during 2013–2015, peaked in 2015, and has since slowed. One third of states did not have any of the five assessed laws. State, local, and territorial strategies to reduce youths’ initiation of e-cigarettes and population exposure to e-cigarette aerosol, including educational initiatives, coupled with federal regulation of tobacco product manufacturing, labeling, and marketing, could help reduce the risks of e-cigarettes on population health, especially among young persons (*2*,*5*).

On October 23, 2017, New York became the ninth state to include e-cigarettes in its comprehensive smoke-free indoor air law.^§^ Thus, one third of the 27 states and DC with comprehensive smoke-free laws that prohibit conventional tobacco smoking in restaurants, worksites, and bars also prohibit e-cigarette use in these venues. Therefore, approximately 75.4% of the U.S. population (an estimated 243.6 million U.S. residents, including 55.7 million children) live in states in which bystanders can be exposed to secondhand e-cigarette aerosol in indoor public spaces. Previous research indicates that one in four U.S. middle and high school students reported past-month exposure to e-cigarette aerosol in a public place in 2015 (*7*). This exposure is of public health concern because the U.S. Surgeon General has concluded that e-cigarette aerosol is not harmless water vapor, and environmental studies have documented harmful and potentially harmful ingredients in secondhand e-cigarette aerosol, including nicotine, heavy metals, ultrafine particulate matter, and volatile organic compounds (*2*). Including e-cigarettes in comprehensive smoke-free laws can prevent involuntary exposures to secondhand e-cigarette aerosol, especially among vulnerable populations such as youths and pregnant women; simplify enforcement of smoke-free policies; and reduce the potential for the renormalization of tobacco product use (*2*).

The remaining types of laws assessed in this study leverage conventional smoking prevention strategies for youths, which have the potential to prevent youths’ e-cigarette access (*2*,*5*). Licensing requirements for tobacco retailers and manufacturers can increase the incentive to comply with tobacco-related laws, including those prohibiting sales to youths (*2*). In addition, restricting self-service tobacco displays can reduce youths’ tobacco access by reducing theft and increasing interactions between customers and retailers (*8*). Increasing the minimum age of tobacco product sales to 21 years is a potential prevention strategy, because 95% of adult smokers begin before age 21, and young adulthood represents a critical period when many smokers progress from experimental to regular tobacco use (*9*). Finally, substantial increases in conventional cigarette prices reduce consumption, especially among youths. To date, data are limited on the impact of e-cigarette taxes on conventional cigarette use; however, similar to conventional cigarettes, e-cigarette price increases would be expected to reduce use by youths (*2*,*5*). Further evaluations of the effectiveness of these strategies can help inform public health practice and planning (*2*,*5*).

FDA is authorized to regulate the manufacturing, sales, distribution, and marketing of tobacco products sold in the United States. In May 2016, the agency asserted jurisdiction over products that meet the definition of a tobacco product, including e-cigarettes. FDA generally cannot restrict public tobacco use, tax tobacco products, or establish a minimum age for tobacco sales above age 18 years (*2*). However, the Family Smoking Prevention and Tobacco Control Act ensures that localities, states, territories, and tribes can continue to play a central role in tobacco prevention and control policies by preserving their authority to regulate sales, marketing, advertising, and use of tobacco products by persons of any age.^¶^ Thus, state, local, territorial, and tribal tobacco control strategies are an important complement to federal regulation, which can help reduce the public health risks of e-cigarettes, particularly among young persons (*2*).

The findings in this report are subject to at least two limitations. First, STATE does not account for local laws, bills under consideration, regulations, opinions of attorneys general, or case law decisions for tobacco control topics other than preemption. For example, at least 400 localities prohibit indoor e-cigarette use and smoking in worksites, restaurants, and bars,** and at least 200 localities have tobacco-21 laws.^††^ Second, statutory requirements and definitions vary across states. For example, although 26 states have laws or regulations prohibiting self-service displays of e-cigarettes, only three of these states (California, Iowa, and New Mexico) prohibit all self-service displays of e-cigarettes; the remaining 21 states restrict self-service displays to adult-only facilities or tobacco specialty stores and vape shops (*6*). Moreover, some states have regulated e-cigarettes by expanding the statutory definition of a tobacco product to include e-cigarettes, regardless of nicotine content, to simplify enforcement (*2*). However, some states define the products as alternative nicotine or vapor products that are exempt from other tobacco product laws, such as licensure requirements and taxes (*2*).

Given that cigarettes and other combusted tobacco products are responsible for the overwhelming burden of tobacco-related death and disease in the United States (*5*), the Surgeon General has recommended actions to uphold and accelerate strategies proven to prevent and reduce combustible tobacco smoking among youths and adults, while simultaneously preventing youths’ use of emerging tobacco products such as e-cigarettes (*2*). A comprehensive tobacco control framework, which includes strategies to prevent all tobacco product use by youths and public exposure to secondhand tobacco smoke and e-cigarette aerosol, is important to protect the public’s health (*2*,*5*).

**Summary**

**What is already known about this topic?**

E-cigarettes are the most commonly used tobacco product among U.S. youths. E-cigarettes typically deliver nicotine, and the U.S. Surgeon General has concluded that nicotine exposure during adolescence can cause addiction and can harm the developing adolescent brain. In addition to federal regulation, states, localities, territories, and tribes maintain broad authority to adopt additional or more stringent requirements regarding tobacco product use, sales, marketing, and other topics.

**What is added by this report?**

As of September 30, 2017, eight states, the District of Columbia (DC), and Puerto Rico prohibited indoor e-cigarette use and smoking in restaurants, bars, and worksites; 26 states prohibited e-cigarette self-service displays; 16 states, DC, and the U.S. Virgin Islands required a retail license to sell e-cigarettes; five states, DC, and Guam had tobacco-21 laws; and eight states, DC, Puerto Rico, and the U.S. Virgin Islands taxed e-cigarettes. Sixteen states have no such laws.

**What are the implications for public health practice?**

State, local, and territorial strategies to reduce youths’ initiation of e-cigarettes and population exposure to e-cigarette aerosol, which include educational initiatives, coupled with federal regulation of tobacco product manufacturing, labelling, and marketing, could help reduce e-cigarettes’ public health risks, especially among young persons.

## Footnotes

* Guam, Puerto Rico, and the U.S. Virgin Islands are the only U.S. territories tracked in STATE System as of June 2017. Additional territories will be added to STATE in the future. https://www.cdc.gov/STATESystem/.

† http://legalsolutions.thomsonreuters.com/law-products/westlaw-legal-research/.

** http://www.no-smoke.org/pdf/ecigslaws.pdf.

†† https://www.tobaccofreekids.org/content/what_we_do/state_local_issues/sales_21/states_localities_MLSA_21.pdf.
